# SARS‐CoV‐2‐associated T‐cell infiltration in the central nervous system

**DOI:** 10.1002/cti2.1487

**Published:** 2024-01-31

**Authors:** Malte Mohme, Christoph Schultheiß, Andras Piffko, Antonia Fitzek, Lisa Paschold, Benjamin Thiele, Klaus Püschel, Markus Glatzel, Manfred Westphal, Katrin Lamszus, Jakob Matschke, Mascha Binder

**Affiliations:** ^1^ Department of Neurosurgery University Medical Centre Hamburg‐Eppendorf Hamburg Germany; ^2^ Medical Oncology University Hospital Basel Basel Switzerland; ^3^ Laboratory of Translational Immuno‐Oncology, Department of Biomedicine University of Basel and University Hospital of Basel Basel Switzerland; ^4^ Department of Legal Medicine University Medical Centre Hamburg‐Eppendorf Hamburg Germany; ^5^ Internal Medicine IV, Oncology/Hematology Martin‐Luther‐University Halle‐Wittenberg Halle (Saale) Germany; ^6^ Hematology and Oncology University Medical Centre Hamburg‐Eppendorf Hamburg Germany; ^7^ Institute of Neuropathology University Medical Centre Hamburg‐Eppendorf Hamburg Germany

**Keywords:** central nervous system, COVID‐19, COVID‐19‐specific T cell, neuroinflammation, SARS‐CoV‐2, TCR sequencing

## Abstract

**Objectives:**

Infection with severe acute respiratory syndrome coronavirus 2 (SARS‐CoV‐2) causes coronavirus disease 2019 (COVID‐19). Although an acute SARS‐CoV‐2 infection mainly presents with respiratory illness, neurologic symptoms and sequelae are increasingly recognised in the long‐term treatment of COVID‐19 patients. The pathophysiology and the neuropathogenesis behind neurologic complications of COVID‐19 remain poorly understood, but mounting evidence points to endothelial dysfunction either directly caused by viral infection or indirectly by inflammatory cytokines, followed by a local immune response that may include virus‐specific T cells. However, the type and role of central nervous system‐infiltrating T cells in COVID‐19 are complex and not fully understood.

**Methods:**

We analysed distinct anatomical brain regions of patients who had deceased as a result of COVID‐19‐associated pneumonia or complications thereof and performed T cell receptor Vβ repertoire sequencing. Clonotypes were analysed for SARS‐CoV‐2 association using public TCR repertoire data.

**Results:**

Our descriptive study demonstrates that SARS‐CoV‐2‐associated T cells are found in almost all brain areas of patients with fatal COVID‐19 courses. The olfactory bulb, medulla and cerebellum were brain regions showing the most SARS‐CoV‐2 specific sequence patterns. Neuropathological workup demonstrated primary CD8^+^ T‐cell infiltration with a perivascular infiltration pattern.

**Conclusion:**

Future research is needed to better define the relationship between T‐cell infiltration and neurological symptoms and its long‐term impact on patients' cognitive and mental health.

## Graphical abstract

In COVID‐19 patients, neurological symptoms and complications are increasingly recognised, but their underlying causes remain poorly understood. This study found that SARS‐CoV‐2‐associated T cells were present in various brain regions of patients who had fatal COVID‐19 pneumonia, with the olfactory bulb, medulla and cerebellum showing the most specific patterns. These T cells primarily consisted of CD8^+^ T cells and were localised around blood vessels. Further research is required to fully understand the connection between T‐cell infiltration in the brain and its impact on patients' cognitive and mental health in the long term.

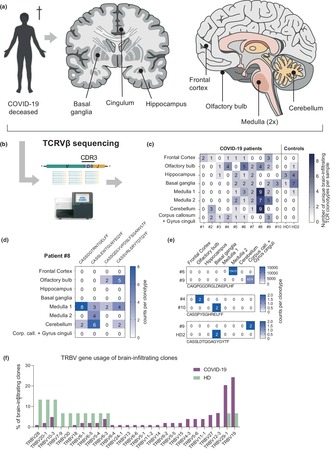

## Introduction

During the severe acute respiratory syndrome coronavirus 2 (SARS‐CoV‐2) pandemic, it has become increasingly evident that coronavirus disease 2019 (COVID‐19) can have neurological implications beyond respiratory symptoms. Many critically ill patients show signs of an acute encephalopathy that is clinically and neuropathologically similar to septic encephalopathy in patients without COVID‐19 and that is probably triggered by SARS‐CoV‐2 infection. COVID‐19 characteristically leads to anosmia and ageusia, which may persist long after the initial infection has resolved.^1^, ^2^ The high incidence of ischemic stroke or cerebral haemorrhage reported in early large observational studies lacking adequate controls has been questioned in recent times. Neuroimmunological complications in COVID‐19 include encephalomyelitis or neuromuscular complications. More prevalent is the occurrence of long‐term COVID‐19, a syndrome primarily characterised by fatigue, sleep disturbances and other symptoms, such as arthralgia and breathlessness. The degree of neuroinflammation and the role of the central nervous system (CNS) inflammation in the complex sequelae summarised as long‐term COVID‐19 is not well understood.^2^, ^3^, ^4^ Evidence suggests that immune‐mediated mechanisms in the CNS play a critical role in post‐infectious sequelae observed in COVID‐19 patients.^5^, ^6^ Although, SARS‐CoV‐2 is not frequently detected in the cerebrospinal fluid (CSF) of patients with neurological manifestations, CSF abnormalities such as lymphocytic pleocytosis and increased antibody production, consistent with encephalitis, are observed.^4^ Interestingly, in a non‐human primate model, SARS‐CoV‐2‐mediated CNS neuroinflammation was observed although the animals did not develop severe respiratory symptoms, indicating an independent pathobiology for neuro‐invasion during COVID‐19.^7^

As SARS‐CoV‐2 infection initiates an adaptive immune response, including virus‐specific T‐cell activation with subsequent CD8^+^ mediated viral clearance of infected cells,^8^, ^9^ we aimed to investigate whether SARS‐CoV‐2‐associated T cells can be detected in distinct regions of the brain. We therefore performed histopathological evaluation as well as T‐cell receptor Vβ sequencing (TRBV) of brain biopsies from patients who deceased after severe COVID‐19 disease.

## Results

### Infiltration patterns of SARS‐CoV‐2 associated T cells in distinct brain regions

**Figure 1:**
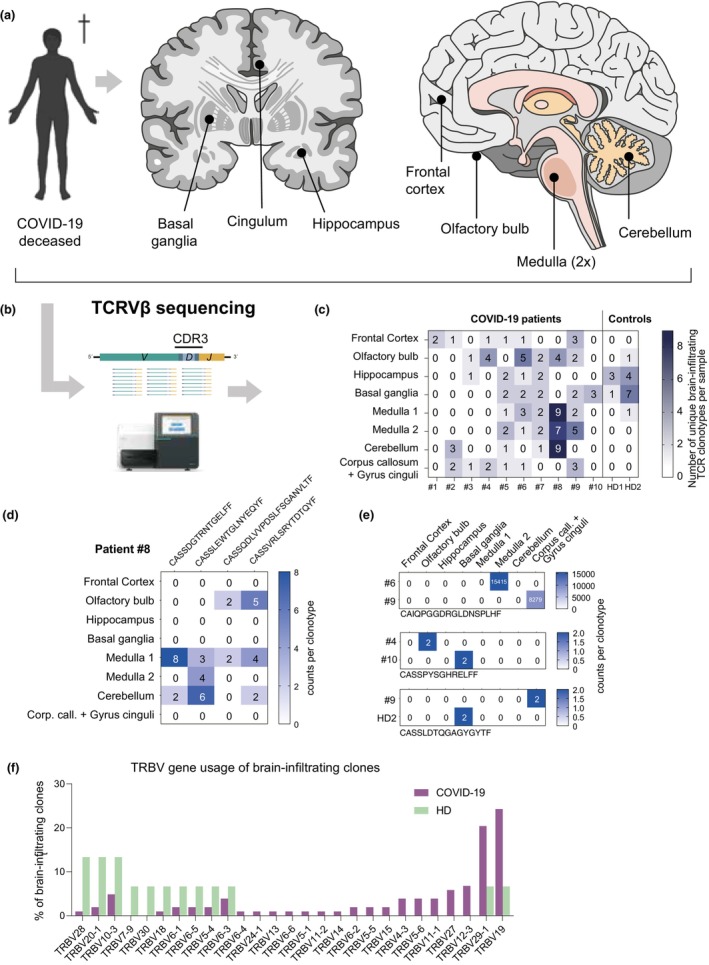
TCR Vβ sequencing of distinct anatomic brain regions in patients deceased from COVID‐19. **(a)** Schematic overview of brain biopsy regions of patients who died from severe SARS‐CoV‐2 virus infections (COVID‐19). **(b)** T‐cell receptor Vβ (TRB) sequencing from gDNA was performed on eight locations. **(c)** Number of brain‐infiltrating T‐cell receptor (TCR) clonotypes per sample of COVID‐19 patients and uninfected controls. One TCR clonotype is defined by a unique CDR3 nucleotide sequence of the rearranged TRB chain. **(d)** Total counts of indicated clonotypes in different brain regions of patient 8. **(e)** Total counts and location of clonotypes shared between individuals. **(f)** TRBV gene usage of brain‐infiltrating TCR clones of COVID‐19 patients and uninfected controls as a bar plot.

The aim of our study was to assess the SARS‐CoV‐2‐specificity and infiltration pattern of brain‐infiltrating T cells in different regions of the brain of patients who deceased from COVID‐19. To this end, we performed the neuropathological autopsy of the brains of patients after severe SARS‐CoV‐2 infection (Supplementary table 1). The brains underwent standard neuropathological workup as described in another previous study.^10^ Specifically, the superior frontal cortex, the hippocampus, the basal ganglia, the corpus callosum with adjacent cingulum, the upper and lower medulla oblongata, the cerebellar hemisphere and the olfactory bulb were studied in detail (Figure 1a and b). Brain tissue from two individuals who deceased from non‐neurological diseases were assessed accordingly as controls. Subsequent TRBV demonstrated oligoclonal infiltration of T cells throughout all analysed brain regions without any hotspot infiltration but a trend towards higher infiltration rates in COVID‐19 patients as compared to the control samples where T cells were only detected in 4 of 8 analysed brain regions (Figure 1c). The most frequently infiltrated areas were the frontal cortex, the olfactory bulb and the corpus callosum with adjacent cingulum (Figure 1c). In total, we detected 103 unique clonotypes in the COVID‐19 specimen and 15 in the control. Patient 8 displayed the highest number of infiltrating clonotypes, especially in the medulla and cerebellum (Figure 1c). From these, four clonotypes were found in multiple brain regions (Figure 1d). In addition, we detected three clonotypes that each were shared between two patient samples or a patient sample and one control individual (Figure 1e). Notably, these shared clonotypes were found in differing brain regions (Figure 1e). All clonotype information including CDR3 amino acid sequence, TRBV(D)J usage and tissue site is summarised in Supplementary tables 2 and 3. Comparing the pan‐tissue TRBV gene usage of brain‐infiltrating clones showed a trend towards preferential usage of TRBV29‐1 and TRBV19 compared to control tissue of non‐SARS‐CoV‐2 infected patients, indicating a skewed T‐cell repertoire, comparable to findings in the peripheral blood of COVID‐19 patients (Figure 1f).^8^

**Figure 2:**
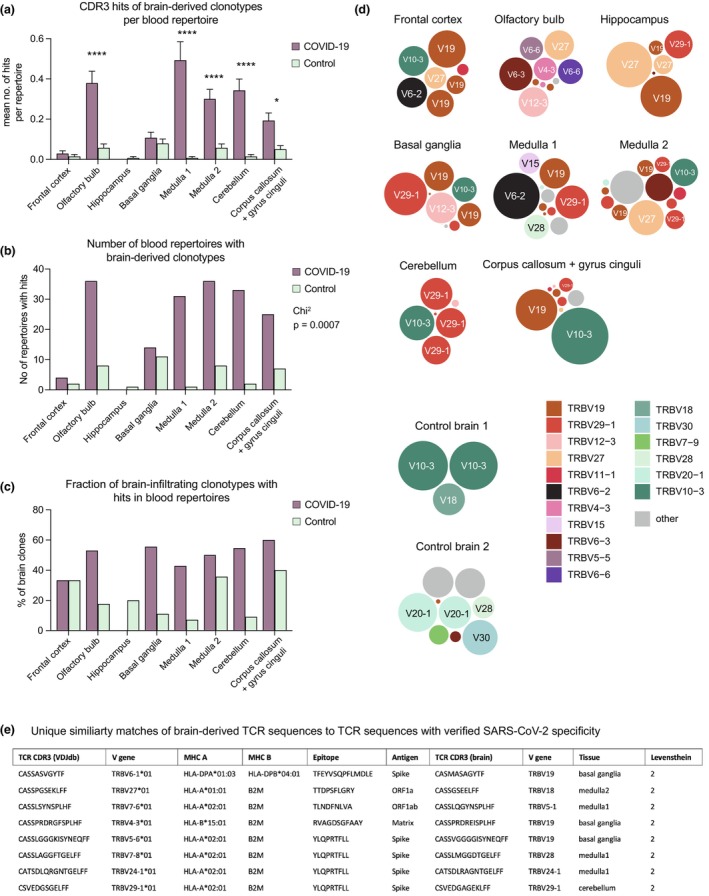
Representation of brain T‐cell clones from deceased COVID‐19 patients across different brain regions. **(a–c)** Search of brain‐infiltrating clones (*n* = 103 clones in total) derived from deceased COVID‐19 patients (*n* = 10 patients) in blood repertoires of unrelated COVID‐19 patients with acute infection (*n* = 140) or in blood of healthy controls (*n* = 140).^8^ The set of 103 brain‐infiltrating clones derived from deceased COVID‐19 patients was divided into eight sets of clones according to the brain area where the clone was detected: Frontal cortex (*n* = 9), olfactory bulb (*n* = 18), hippocampus (*n* = 6), basal ganglia (*n* = 11), medulla 1 (*n* = 17), medulla 2 (*n* = 18), cerebellum (*n* = 14), corpus callosum and gyrus cinguli (*n* = 10). A hit is considered a TCR clone with an identical CDR3 amino acid sequence of the rearranged TRB chain. **(a)** Mean hits per repertoire. Error bars represent SEM. * and **** indicate *P*‐values < 0.05 and < 0.0001, two‐way ANOVA. **(b)** Number of blood TCR repertoires which contained at least one of the brain‐infiltrating clones of the corresponding set. **(c)** Percentage of brain‐infiltrating clones derived from deceased COVID‐19 patients which were found in at least one blood TCR repertoire of unrelated COVID‐19 patients or healthy controls. **(d)** Bubble plots of brain‐infiltrating TRB clones derived from 10 deceased COVID‐19 patients grouped by brain area of origin. Brain repertoires of two uninfected control patients are shown as a comparison. One bubble represents one clone, which is defined by a unique CDR3 nucleotide sequence of the TRB chain. The area size of the bubbles corresponds to the clonal fraction within the repertoires. TRBV genes are coded by fill colour. **(e)** Listing of brain‐derived clonotypes that showed unique similarity matches (based on indicated Levenshtein distance) to CDR3 sequences derived from the VDJdb database with verified SARS‐CoV‐2‐reactive TCRs (*n* = 5609).

Although minor T‐cell infiltrates in the brain contribute to the maintenance of CNS homeostasis,^11^ generally brain‐infiltrating T cells are predominantly associated with inflammation and pathology. We postulated that the 103 clonotypes identified in all specified brain regions of the 10 deceased COVID‐19 patients may exhibit a higher abundance of SARS‐CoV‐2‐associated T cells. To validate our hypothesis, we examined the presence of these clonotypes in the blood repertoires of unrelated COVID‐19 patients with active infection (*n* = 140; median age 49 years, range 8–85 years, 46% female, 54% male, 740 000 unique TCR clonotypes) and blood samples from healthy individuals (*n* = 140; median age 42 years, range 10–87 years, 49% female, 51% male, 650 000 unique TCR clonotypes) from our previously published repository.^8^ We found that the relative number of exact hits per blood repertoire as well as the total amount of blood repertoires with an exact hit was substantially higher for COVID‐19 patients when the tested clonotypes were derived from the olfactory bulb, medulla, cerebellum and corpus callosum including gyrus cinguli (Figure 2a and b). Notably, more than half of the clonotypes derived from these brain regions showed exact hits in the peripheral repertoires of COVID‐19 patients suggesting that these SARS‐CoV‐2‐associated T cells have a tissue tropism towards these brain areas (Figure 2c). To further assess and illustrate these T‐cell clonality patterns, we plotted all brain‐infiltrating T‐cell clones according to their size, TRBV gene usage and brain area (Figure 2d). These data clearly demonstrate the skewed distribution of TRBV gene usage among these clones (Figure 1d). Next, we mined 5609 SARS‐CoV‐2‐reactive TRBV clonotypes deposited in the VDJdb database for matches to our brain‐derived dataset. While there were no exact CDR3 matches, we detected a total of 192 similarity matches when applying Levensthein distance 1 (for 3/192, all brain‐derived) or 2 (for 189/192; Supplementary table 4). Also, in 16/192 cases, the TRBV gene assignments matched. Most hits in our COVID‐19 cohort were generated from clonotypes with similarity towards validated SARS‐CoV‐2 spike (44%), ORF1ab (21%), NCP (17%) and NSP3 (10%) reactive T‐cell clones (Supplementary table 4). Notably, sequences related to anti‐spike reactivity were dominant in the medulla and cerebellum (Supplementary table 4). From the total of 192 matches, 170 matches were generated from 38 unique brain‐derived sequences to 156 unique VDJdb‐derived sequences (Supplementary table 4). From these 38 sequences, eight generated single matches to verified clonotypes from the database (Figure 2e). Half of these matches map to sequences targeting the HLA‐A*02:01‐restricted YLQPRTFLL epitope of the spike protein (Figure 2e). Interestingly, the CATSDLRAGNTGELFF and CSVEDGAGEKLFF derived from patient 8 also map to the identical TRBV gene as the verified sequence (TRBV24‐1 and TRBV29‐1, respectively; Figure 2e). In addition, the medulla 2‐derived clonotype CASRPANTGELFF (TRBV27) generates similarity matches with 20 different clonotypes that target the YLQPRTFLL epitope of the spike protein in the HLA‐A*02:01 context. Using Levenshtein distance 2, we also detected 22 hits generated by six unique clonotypes from the control samples. From these 22 hits, one clonotype accounts for 14 hits with clonotypes that target five different SARS‐CoV‐2 epitopes arguing for the non‐specificity of the findings.

### Morphological assessment of distinct brain regions for reactive and inflammatory changes

**Figure 3:**
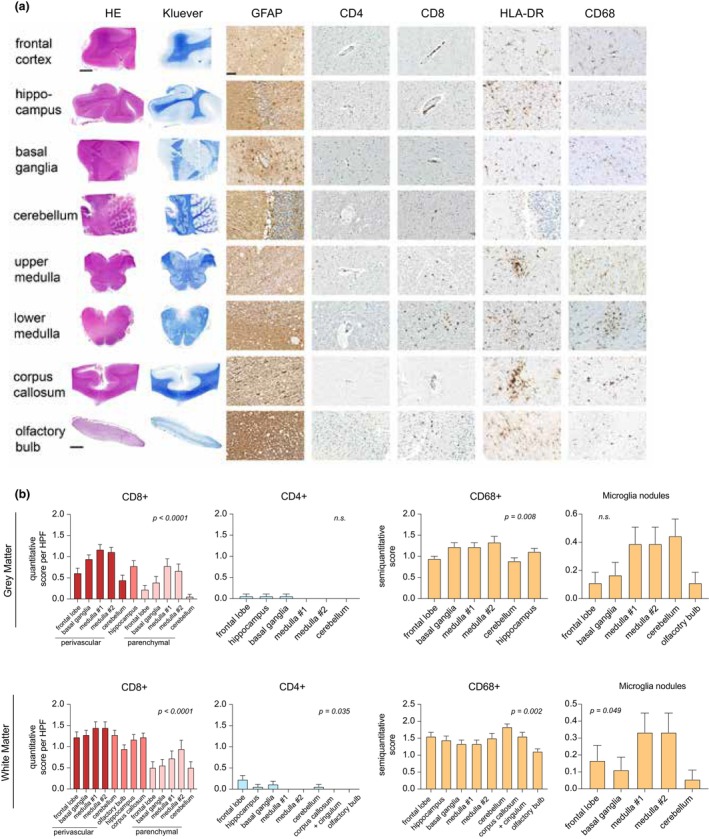
Histopathological and immunohistochemical analysis of different brain regions from COVID‐19 deceased patients. **(a)** Haematoxylin & eosin‐ (HE) and Kluever stainings to assess general morphology and myelination status (first two columns). The remaining 5 columns show the results of the immunohistochemistry for GFAP, CD4^+^ and CD8^+^ T cells, MHC class II expression (HLA‐DR) and microglia/macrophage infiltration (CD68) to assess the degree of immune infiltration and local neuroinflammation. In comparison with the hindbrain, reactive astrogliosis was more pronounced in the forebrain. Generally, perivascular CD4^+^ cells were very sparse, while both perivascular and parenchymal CD8^+^ cells were seen more frequently, especially in the medulla oblongata. The degree of microgliosis varied both between patients and regions, changing between diffuse patterns and microglial nodules (see HLA‐DR of upper medulla and corpus callosum, as well as CD68 of lower medulla). **(b)** Subsequent semiquantitative analysis of immune cell infiltration and astrogliosis in grey and white matter. For statistical testing, ANOVA was used.

To better understand the phenotype of COVID‐19‐related T‐cell infiltration and to analyse whether different T‐cell subsets (i.e. CD4^+^ or CD8^+^) distinctively infiltrate in different brain regions, we performed immunohistochemical analysis for the most prevalent immune cell populations, including, CD4^+^, CD8^+^ T cells and CD68^+^ microglia/macrophages (Figure 3). As a whole, CD4^+^ T lymphocytes were only very scarce, while both CD8^+^ T lymphocytes and activated microglia could be found mostly in the white matter of the lower brainstem, a finding we have described previously^10^ (Figure 3a and b). Both CD4^+^ and CD8^+^ T lymphocytes were more frequently situated in the perivascular spaces (Figure 3). The more prevalent CD8^+^ T‐cell infiltration is in line with a virus‐specific immune response with CD8^+^ cytotoxic T cells. Microglia nodules, which indicate an inflammatory response and microglia activation, were mainly seen in the analysed regions of the brainstem (Figure 3a and b). The degree of reactive astrogliosis as reflected by immunohistochemistry for glial fibrillary acidic protein (GFAP) tended to be more pronounced in the white matter when compared with the grey matter – a finding which parallels the experience from daily neuropathological diagnostic work and which we have demonstrated to be present in COVID‐19 patients and controls (Figure 3 and Supplementary figure 1).^10^ When looking only at grey matter, the allo‐ and isocortex as well as the central grey matter appeared to be more gliotic compared to the hindbrain. Small foci of demyelinisation were rare.

## Discussion

SARS‐CoV‐2, the virus responsible for the COVID‐19 pandemic, primarily affects the respiratory system. However, there is increasing evidence that the virus also leads to neuroinflammation causing neurological symptoms such as headache, cognitive disturbances and even more severe symptoms such as seizures, encephalitis and stroke.^4^, ^12^

Several high‐quality autopsy‐based studies of patients who succumbed to severe SARS‐CoV‐2 infections led to the concept of vascular damage and subsequent perivascular inflammation with disruption of the blood–brain barrier as the favoured explanation for the neurologic symptoms associated with SARS‐CoV‐2 infection.^13^, ^14^ Two studies so far reported conflicting results with potential evidence of direct neuronal infection of the virus.^15^, ^16^ However, overt signs of diffuse encephalitis were missing highlighted by the absence of deeper brain parenchyma infiltrating T‐ and other inflammatory cells, questioning the validity of these findings.^17^, ^18^, ^19^ The latest research rather points towards an infection of non‐neuronal cells.^20^

In concordance with the finding of endothelial damage found in other organs,^21^ most studies support endothelitis with subsequent microglial activation and T‐cell infiltration caused either directly by endothelial SARS‐CoV‐2 infection or indirectly by paracrine effects of inflammatory cytokines on the endothelium as a uniform sign of neuroinflammation in COVID‐19.^19^, ^22^, ^23^ However, it is important to note that the role of these CNS infiltrating T cells is not yet fully understood and so far, no SARS‐CoV‐2‐specific T cells were confirmed in the CNS of COVID‐19 patients.

Although an adaptive T‐cell response is essential to control and clear viral infections, it can also initiate and maintain chronic inflammation.^24^ Therefore, it is currently unclear whether the infiltration of SARS‐CoV‐2‐specific T cells in the perivascular compartment of the brain is a cause or a consequence of a severe SARS‐CoV‐2 infection leading to the observed associated neurological symptoms. Another aspect that needs to be considered in the future is the potential cross‐reactivity of virus‐specific T‐cell activation. In autoimmune diseases, such as multiple sclerosis, a transient increase of Epstein–Barr virus (EBV) antibody titres was associated with a higher risk for a disease onset, indicating that misguided T‐cell reactivity could be initiated as a result of temporary viral infections. This concept was initially described as ‘molecular mimicry’.^25^ Our detection of SARS‐CoV‐2‐associated T‐cell clonotypes in the brain of severely infected patients therefore points to either ongoing SARS‐CoV‐2 antigen presentation, persistent infiltration of formerly activated virus‐specific T cells or, in rare circumstances, the recognition of a potential molecular mimicry antigen. It should be noted that the here reported enrichment patterns of shared TCR sequences originating from different COVID‐19 patients as well as detected sequence similarity matches with experimentally validated T‐cell clonotypes from public repository not unambiguously indicate specificity for SARS‐CoV‐2 antigens. While TCR sequences can converge to different CDR3 motifs when targeting the same antigen, single amino acid variations in otherwise identical CDR3 sequences often alter – also dependent on the topological position – binding specificities completely.^26^ These TCRs require further functional validation. In addition, our study is limited by the scarcity of ‘healthy’ controls because of the heterogeneous nature of autopsy patients and by the concurrent neurological diseases in available post‐mortem donors.

In conclusion, given the tremendous number of people infected with SARS‐CoV‐2 worldwide, with potential long‐term sequelae of concomitant neuroinflammation, the role of CNS‐infiltrating and bona‐fide virus‐specific T cells represents an important area for future research. With the application of a not yet employed methodology, our study provides strong evidence for SARS‐CoV‐2‐specific T‐cell infiltration into the brain in response to infection with the virus. Nevertheless, the small cohort size and thus a relatively small number of recovered clonotypes together with a lack of HLA typing requires confirmation of SARS‐CoV‐2 specificity in future studies. Although the precise role of T cells in COVID‐19‐associated neurological symptoms is not fully understood, future studies should aim to elucidate the mechanisms of reactive neuroinflammation in relation to virus‐specific T‐cell infiltration during viral infections, the risk of CNS autoimmunity and the relationship between T‐cell infiltration and neurological symptoms in patients with COVID‐19. This will help to better define the neuropathological consequences of COVID‐19 and their potential long‐term impacts on patients' cognitive and mental health.

## Methods

### Study population

We analysed 10 patients who died from direct sequelae of a SARS‐CoV‐2 infection from 03/2020 to 09/2020 using TCR sequencing. An additional eight patients were analysed immunohistochemically. Patients were presumably infected with the SARS‐CoV‐2 D614G variant, which was the most prevalent strain in the first wave in Germany.^27^ All patients had confirmed SARS‐CoV‐2 infection by throat swab followed by immediate quantitative RT‐PCR analysis prior to autopsy. Autopsies were performed according to § 25 of the German Infection Protection Law (IfSchG). Two non‐COVID‐19 patients who died from other, non‐neurological diseases were used as controls. The use of human tissue for post‐mortem studies has been approved by the institutional review board of the independent ethics committee of the Hamburg Chamber of Physicians (PV7311 and 2020‐10 353‐BO‐ff). The study complied with the tenets of the Declaration of Helsinki.

### Tissue sampling and histopathological evaluation

In the federal state of Hamburg, Germany, all persons who died as a result of or with an infection of SARS‐CoV‐2 during the period from 03/2020 to 12/2020 underwent full autopsy at the Institute of Legal Medicine of the University Medical Centre of Hamburg‐Eppendorf (UKE). After removal during autopsy, brains were fixed in buffered 4% formaldehyde, examined macroscopically and subsequently underwent extensive neuropathological workup at the Institute of Neuropathology of the UKE. Formalin‐fixed paraffin‐embedded tissue samples from the superior frontal gyrus, hippocampus, basal ganglia, corpus callosum with adjacent cingulum, upper and lower medulla oblongata and cerebellar hemisphere (Figure 1a) were processed and stained with haematoxylin and eosin following standard laboratory procedures. For the integrity assessment of the myelin sheaths, Kluever's stain was performed. Furthermore, immunohistochemistry with antibodies to human glial fibrillary acidic protein (GFAP; 1:200, clone 6F2; Agilent, Santa Clara, USA), human leukocyte antigen DR, (HLA‐DP, DQ and DR; 1:200, mouse clone CR3/43; Agilent), cluster of differentiation 68 (CD68; 1:100, clone PG‐M1, Agilent) and human cluster of differentiation 8 (CD8; 1:100, clone SP239, Spring Bioscience, Pleasanton, USA) was performed on a Ventana benchmark XT autostainer following the manufacturer's recommendations. The quality of the immunohistochemical stains was assessed by on‐slide positive controls for all antibodies. The degree of astrogliosis and microgliosis was quantified using a four‐tiered semiquantitative approach for GFAP and HLA DR (none, slight, moderate and severe). For quantitative assessment of infiltration with CD8^+^ and CD4^+^ lymphocytes, positively stained cells were counted per 0.5 mm^2^ (HPF). Infiltration was categorised as none, mild (1–9 cells per HPF), moderate (10–49 cells per HPF), or severe (≥ 50 cells per HPF). In addition, genomic DNA was isolated for TCR Vβ sequencing using standard protocols.

### TCR Vβ sequencing

The T‐cell receptor (TCR) repertoire of brain‐infiltrating T cells was assessed using next‐generation sequencing (NGS) of the TRB genetic locus. In brief, genetic loci were amplified together in a multiplex PCR using TRB‐A/B primer pools and 500 ng of genomic DNA.^28^ The primers were purchased from Metabion International AG (Martinsried, Germany). Two consecutive PCRs were performed to generate fragments tagged with illumina‐compatible adapters for hybridisation to the flow cell and 7 nucleotide barcodes for sample identification. All PCRs were performed using Phusion HS II (Thermo Fisher Scientific Inc., Darmstadt, Germany). After gel electrophoretic separation, amplicons were purified using the ‘NucleoSpin’ Gel and PCR Clean‐up kit (Macherey‐Nagel, Düren, Germany), quantified on the Qubit platform (QIAGEN, Hilden, Germany) and pooled to a final concentration of 4 nM. The quality of the amplicon pools was controlled on an Agilent 2100 Bioanalyzer (Agilent Technologies, Böblingen, Germany) before undergoing NGS on an Illumina MiSeq (paired‐end, 2 9301‐cycles). Samples were sequenced at an average sequencing depth of 56 000 reads. Annotation of TRB loci rearrangements was computed with the MiXCR framework (3.0.8).^29^ As a reference for sequence alignment, the default MiXCR library was used. Non‐productive reads and sequences with fewer than 2 read counts were not considered for further analysis. Each unique complementarity‐determining region 3 (CDR3) nucleotide sequence was defined as one clone.

Brain‐derived clones were searched based on exact CDR3 amino acid sequence identity in 140 blood T‐cell repertoires from uninfected controls or 140 repertoires from patients with active COVID‐19 using R version 3.6.3. Bubble plots were computed using R packages packcircles and ggplot2.

### Statistical analysis

Data were analysed using R version 3.6.3 and the packages tcR, ade4 and tidyverse (tidyverse.org).^30^, ^31^ Graphs were plotted using Adobe Illustrator 2023 and GraphPad Prism 9.5.1.

## Author contributions

**Malte Mohme:** Conceptualization; data curation; formal analysis; investigation; methodology; project administration; resources; supervision; visualization; writing – original draft; writing – review and editing. **Christoph Schultheiß:** Data curation; formal analysis; investigation; methodology; resources; software; supervision; validation; visualization; writing – original draft; writing – review and editing. **Andras Piffko:** Data curation; formal analysis; investigation; validation; writing – original draft. **Antonia Fitzek:** Data curation; project administration; resources. **Lisa Paschold:** Data curation; formal analysis; software. **Benjamin Thiele:** Data curation; formal analysis; supervision. **Klaus Püschel:** Conceptualization; funding acquisition; project administration; supervision. **Markus Glatzel:** Resources; supervision. **Manfred Westphal:** Resources; supervision. **Katrin Lamszus:** Project administration; resources; supervision. **Jakob Matschke:** Conceptualization; data curation; formal analysis; investigation; methodology; resources; validation; writing – original draft; writing – review and editing. **Mascha Binder:** Conceptualization; data curation; formal analysis; funding acquisition; methodology; supervision; writing – original draft; writing – review and editing.

## Conflict of interest

The authors declare no conflict of interest.

## Supporting information


Supplementary tables 1‐4

Supplementary figure 1

## Data Availability

The data that support the findings of this study are available from the corresponding author upon reasonable request. Sequencing data are deposited at ENA under the accession number PRJEB67800.
