# Supplementary material for: SARS‐CoV‐2‐associated T‐cell infiltration in the central nervous system
Source: Clin Transl Immunology. 2024 Jan 31;13(2):e1487. doi: 10.1002/cti2.1487 (PMC10831126; doi:10.1002/cti2.1487)
Supplement: Supplementary file 2 — Supplementary figure 1 [file CTI2-13-e1487-s001.docx]

 Histopathological analysis of different brain regions from COVID-19 deceased patients. Semiquantitative analysis of the immunohistochemistry for GFAP **(a)**and MHC class II expression (HLA-DR) **(b)** to assess the degree of astrogliosis and immune activation/neuroinflammation. Statistical testing was performed with ANOVA.
